# Development, Validation, and Application of an HPLC Method Combined with an In Vitro Model for the Determination of Antibiotic Binding to the Haemoadsorber CytoSorb^®^

**DOI:** 10.3390/molecules31132337

**Published:** 2026-07-03

**Authors:** Sara Kenda, Jakob Gubenšek, Tomaž Vovk

**Affiliations:** 1 Faculty of Pharmacy, University of Ljubljana, Askerceva 7, 1000 Ljubljana, Slovenia; sara.kenda@sbng.si; 2 General Hospital Dr. Franc Derganc Nova Gorica, Padlih Borcev 13a, 5290 Sempeter pri Gorici, Slovenia; 3 Department of Nephrology, University Medical Center Ljubljana, Zaloska 7, 1000 Ljubljana, Slovenia; jakob.gubensek@kclj.si; 4 Faculty of Medicine, University of Ljubljana, Vrazov trg 2, 1000 Ljubljana, Slovenia

**Keywords:** antibiotics, critically ill, CytoSorb^®^, gradient elution, haemoadsorption, hemoadsorption, in vitro model

## Abstract

Supportive therapy with haemoadsorption is gaining popularity in critically ill patients, with the aim of reducing overinflammation triggered by the cytokine storm. The haemoadsorbers used are not specific for cytokines and also bind antibiotics. The aim of this study was to develop and validate a simple analytical method for the simultaneous determination of selected antibiotics and to develop an in vitro model for the quantification of their binding to the CytoSorb^®^ haemoadsorber under conditions simulating sepsis. Imipenem (IMI), amoxicillin (AMO), cefepime (CEF), meropenem (MERO), vancomycin (VAN) and piperacillin (PIP) were measured in bovine plasma via precipitation with acetonitrile and liquid–liquid extraction with dichloromethane. The aqueous phase was collected and analysed using a C18 HPLC system under gradient conditions, with modulation of organic solvent content and mobile phase pH, and detection performed using a UV/Vis detector. The method was linear (r^2^ > 0.982) across investigated analytical ranges (1.0–100.0 µg/mL for AMO and VAN, 1.0–75.0 µg/mL for CEF, MERO and PIP and 2.5–100.0 µg/mL for IMI). Intra- and inter-day precision did not exceed 14% and accuracy ranged from 85.8% to 108.5%. Using the in vitro model, we showed that CytoSorb^®^ significantly removed VAN and PIP, but not MERO. Further clinical studies are needed to establish the clinical significance of these findings and their impact on antibiotic exposure.

## 1. Introduction

Antibiotics are widely used drugs, and their use in intensive care units (ICUs) can be up to ten times higher than in other departments [1]. Up to 64% of patients treated in the ICU receive antibiotics [2]. The pharmacokinetic and pharmacodynamic issues are altered in critically ill patients, making therapy optimisation very difficult [1]. At the pharmacokinetic level, the greatest changes occur in the volume of distribution and drug clearance. The main reasons for the altered volume of distribution are (i) altered capillary permeability, which increases the interstitial space; (ii) large quantities of intravenous fluids, which increase the volume of distribution of hydrophilic drugs; (iii) hypoalbuminemia, which mainly affects drugs that are highly bound to plasma proteins. Most antibiotics are excreted through the kidneys, and critically ill patients may experience either increased or decreased excretion of antibiotics [1,3]. Antibiotics have different pharmacokinetic/pharmacodynamic (PK/PD) targets, but because of all these changes, it is difficult to achieve them in critically ill patients [1]. Moreover, critically ill patients may also be treated with dialysis, extracorporeal membrane oxygenation (ECMO), or even haemoadsorption, which can make drug dosing even more difficult.

Rapid reduction in inflammatory mediators in patients with sepsis is essential for achieving improved circulatory stability and, consequently, better survival. Recently, the CytoSorb^®^ haemoadsorber has been used for the purpose of extracorporeal elimination. It is a porous, biocompatible polymer with properties that enable it to bind many hydrophobic molecules with a molecular weight of up to 55 kDa, which includes most cytokines. It removes these molecules based on size and hydrophobic interactions, and the removal is concentration-dependent. The haemoadsorber can be used alone or in combination with renal replacement therapy, cardiopulmonary bypass, or ECMO. Treatment duration ranges from 6 h to 7 days, with the manufacturer recommending that the haemoadsorber be changed every 24 h [4]. CytoSorb^®^ binds cytokines released during sepsis, as well as free haemoglobin, myoglobin, bilirubin, bile acids, bioactive lipids, light chains of immunoglobulins, some toxins, bacterial endotoxins, and other toxic metabolites from the blood. In addition, the haemoadsorber can also affect the removal of drugs from the blood [5]. In vitro studies have shown that vancomycin, teicoplanin, and aminoglycosides are partially or completely removed [6]. There is limited information on optimising antibiotic dosing in patients with sepsis, treated with haemoadsorption [7]. It should be noted that CytoSorb^®^ is also used to treat poisoning with drugs such as ticagrelor and venlafaksine [8,9,10].

Current analytical methods for the determination of antibiotics in biological samples primarily rely on LC-UV/Vis and LC-MS/MS techniques; however, LC-MS/MS remains limited to specialised laboratories and is not yet routinely available in all clinical settings. Most chromatographical analytical methods developed to date have analysed only two or three antibiotics simultaneously, which was insufficient for our purposes [11,12,13,14,15]. Some methods have been developed to determine six or even twelve antibiotics simultaneously, but none included all the antibiotics required for our study [11,15]. The aim of this study was to develop and validate a simple analytical method for the simultaneous determination of the most commonly used antibiotics in ICU therapy, namely imipenem (IMI), amoxicillin (AMO), cefepime (CEF), meropenem (MERO), vancomycin (VAN), and piperacillin (PIP). The method was specifically developed and validated for application in an in vitro study investigating antibiotic binding to the CytoSorb^®^ haemoadsorber using bovine blood. Furthermore, an in vitro model was developed to evaluate drug binding to the CytoSorb^®^ haemoadsorber and was applied to selected antibiotics.

## 2. Results and Discussion

### 2.1. Development of Sample Preparation Method

We tested three different methods of sample preparation: (i) protein precipitation with acetonitrile (ACN) (plasma and working solutions: ACN, 1:3 *v*/*v*), drying and reconstitution, (ii) protein precipitation with HClO_4_ followed by extraction in ethyl acetate, and (iii) protein precipitation with ACN (plasma and working solutions: ACN, 1:1 *v*/*v*) and extraction in dichloromethane. The baseline method was protein precipitation with ACN, a drying and reconstitution method, which resulted in poor accuracy at low concentrations and nonlinearity across the concentration range. We found that analyte losses occurred during the drying process, which were particularly pronounced at low concentrations. To overcome reduced analyte response and interfering peaks, we decided to combine protein precipitation with an additional clean-up step using organic solvents [16]. We used a method involving protein precipitation with HClO_4_ and extraction in ethyl acetate followed by direct analyses of aqueous phase. However, this method was also ineffective, as it caused a loss of peak shape and reduced analyte responses in the chromatogram. The final method, with protein precipitation with ACN, extraction in dichloromethane and direct analysis of aqueous phase, enabled good separation of analytes and appropriate sensitivity to a rich target concentration range. In previously published studies analysing six and twelve antibiotics, respectively, sample preparation was performed using protein precipitation and dilution [11,15], which represents simpler approaches compared to our method. The addition clean-up step included in our method most likely influenced the analytical range, as lower concentrations of most studied antibiotics could be determined compared with the literature methods [11,15]. Furthermore, although the sample preparation is more complex, the analytical run time of our method is shorter by 5–7 min compared to reported methods [11,15], thereby increasing sample throughput. We therefore conclude that developed sample preparation improves analytical selectivity, enabling reliable analyte quantification, and represents a comparable alternative for multi-analyte determination.

### 2.2. Development of Chromatographic Method

**Figure 1:**
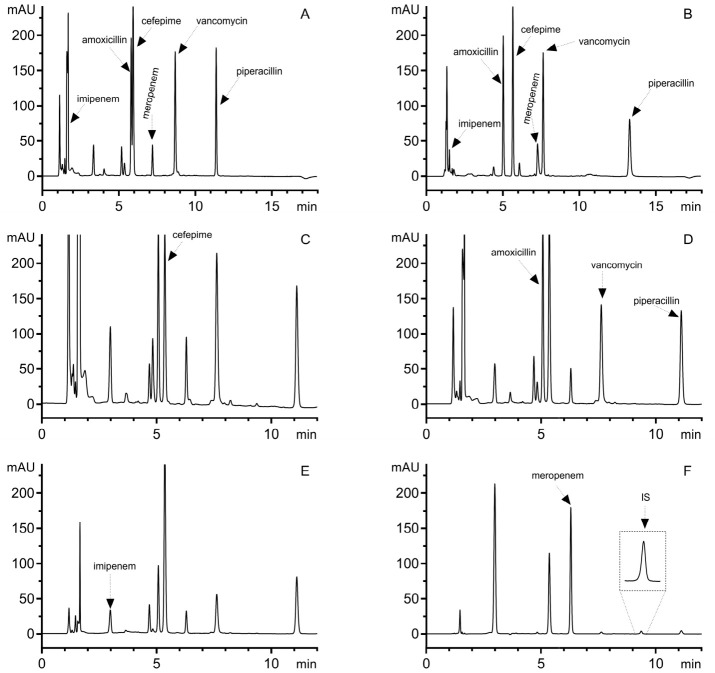
Chromatographic method development and optimisation for the determination of selected antibiotics in plasma. (**A**) Gradient elution at mobile phase pH 7.0; (**B**) gradient elution at pH 3.0; (**C**–**F**) final method based on a combined organic solvent and pH gradient. Detection was performed at 220 nm for cefepime (**C**), 230 nm for amoxicillin, piperacillin, and vancomycin (**D**), 245 nm for imipenem (**E**), and 300 nm for meropenem and the internal standard (**F**). Plasma samples were spiked at 100 µg/mL.

Several reversed-phase analytical columns (Eclipse XDB C18 (4.6 × 150 mm, 5 μm), Eclipse XDB C8 (4.6 × 150 mm, 5 μm), Waters XTerra RP18 (4.6 × 150 mm, 5 μm), Phenomenex Luna C18 (4.6 × 150 mm, 3 μm) and mobile phases (0.5% H_3_PO_4_ at pH 7.0 with ACN (MP1), 0.5% H_3_PO_4_ at pH 3.0 with ACN (MP2), A: 0.5% H_3_PO_4_ at pH 7.0, B: ACN + 0.5% H_3_PO_4_ at pH 3.0 (1:3, *v*/*v*) (MP3)), were tested to optimise the retention time, chromatographic peak shape, width and separation of IMI, AMO, CEF, MERO, VAN and PIP from endogenous peaks and internal standard (IS). Optimal separation was achieved on the Eclipse XDB C18 using MP3 under pH gradient mobile phase (Figure 1). With other stationary phases there was no separation of analytes, a poor baseline, asymmetrical chromatographic peaks, and low analyte responses.

No separation between AMO and CEF was observed with gradient mobile phase with pH 7.0 (Figure 1A), and no adequate separation between MERO and VAN was observed with gradient mobile phase with pH 3.0 (Figure 1B). Furthermore, with pH 3.0, the responses were only half as large as with pH 7.0. Also, the time taken to separate peaks became longer. After reviewing the results obtained with pH 7.0 and pH 3.0 and the literature [17], we realised that we could influence the separation of the chromatographic peaks by changing the pH of the mobile phases. We decided to use a pH gradient as well as gradient of organic solvent (MP3). The final method was obtained by optimising both gradients—organic solvent composition and mobile phase pH—as well as the detection wavelengths, resulting in adequate analyte separation without co-elution, short run time, and appropriate sensitivity. Figure 1C–F present the final separation method at different wavelengths, where we achieved the best responses, optimal peak separation, and appropriate accuracy and repeatability. Figure 1C shows the chromatographic peak for CEF at a wavelength of 220 nm; Figure 1D shows the separation of AMO, VAN and PIP at 230 nm; Figure 1E shows the separation of IMI at 245 nm; and Figure 1F shows the separation of MERO and IS (with magnification). Chromatograms at the lower limit of quantification are provided in the  Supplementary Material (Figure S1). The use of a pH gradient represents a key methodological advantage, enabling a substantial reduction in run time compared with isocratic conditions at pH 3.0 (~14 min).

### 2.3. Method Validation

#### 2.3.1. Linearity, Lower Limit of Quantification and Concentration Range

The developed method was aimed to cover the analytes reference ranges. The reference range for VAN is 10 to 20 µg/mL, but to determine the effectiveness of the treatment, it is necessary to consider the area under the curve (AUC), which should be between 400 and 600 mg·h/L [18]. IMI, AMO, CEF, MERO and PIP are beta-lactam antibiotics with time-dependent PK/PD targets. The effectiveness of time-dependent antibiotics is defined as the time in which free concentration is above the minimal inhibitory concentration (MIC). Therapeutic monitoring of plasma concentrations of beta-lactam antibiotics is rare, limited to research purposes and individual institutions. As a result, and due to the wide spectrum of pathogens they treat, accurate reference values are poorly established and usually very broad [19]. The developed method is linear from 1.0 to 75.0 µg/mL for CEF, MERO and PIP, from 1.0 to 100.0 µg/mL for AMO and VAN, and from 2.5 to 100.0 µg/mL for IMI. The coefficient of determination ranged from 0.982 to 1.000. The lower limit of quantification (LLOQ) was 1.0 µg/mL for AMO, CEF, MERO, VAN and PIP, and 2.5 µg/mL for IMI (Table 1). Similar results were obtained in human plasma as in bovine plasma (Supplementary Material, Table S1), indicating promising initial performance; however, additional validation studies are required before the method can be applied to human samples.

The obtained method ranges and sensitivity are comparable with the literature where the simultaneous determination of at least six or more antibiotics was used [11,15]. 

#### 2.3.2. Precision and Accuracy

The obtained intra- and inter-day precision of selected antibiotics was within the target limits, with values not exceeding 14.0% for intra-day and 14.3% for inter-day precision, respectively. The accuracy ranged between 85.8 and 109% for all of the analytes (Table 1).

#### 2.3.3. Selectivity and Carry-Over

**Figure 2:**
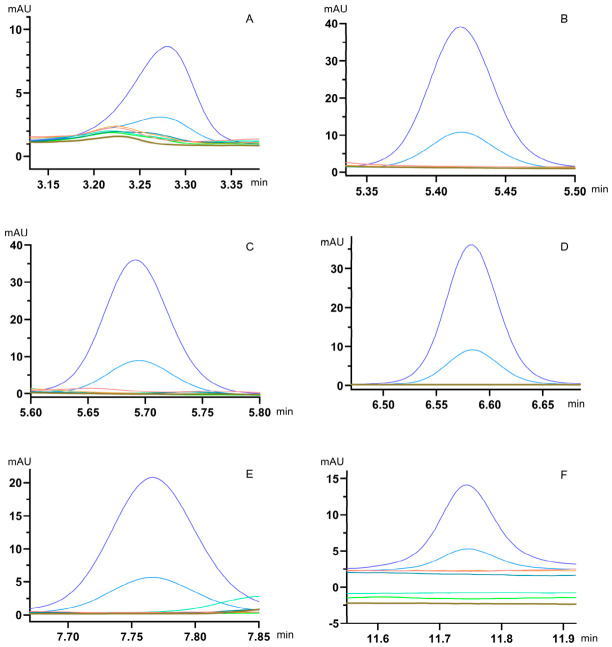
Selectivity assessment of the developed HPLC–UV method. Chromatograms of six individual blank plasma samples and plasma samples spiked with antibiotics at 2.5 µg/mL and 10 µg/mL are presented for (**A**) imipenem (245 nm), (**B**) amoxicillin (230 nm), (**C**) cefepime (220 nm), (**D**) meropenem (300 nm), (**E**) vancomycin (230 nm), and (**F**) piperacillin (230 nm).

The selectivity study demonstrated the absence of interfering peaks in blank plasma samples at the retention times of analytes and IS (Figure 2). Additionally, no carry-over was observed.

#### 2.3.4. Freeze–Thaw, Long-Term and Postpreparative Stability

The freeze–thaw stability study showed that the measured concentrations for IMI, AMO, CEF, MER, VAN, and PIP ranged from 94.0 to 109.6%, 89.6–99.9%, 94.5–99.9%, 92.9–102.6%, 86.3–96.3%, and 93.0–99.2% of nominal values, respectively, indicating that freeze–thaw cycles did not significantly affect analyte concentrations.

Long-term stability, evaluated over one week at −70 °C, confirmed that the antibiotics remained stable under the applied storage conditions. The measured concentrations for IMI, AMO, CEF, MER, VAN, and PIP ranged from 92.4 to 97.5%, 87.2–103.2%, 104.8–106.2%, 84.1–91.6%, 93.1–103.9%, and 108.4–110.1% of nominal values, respectively. 

**Figure 3:**
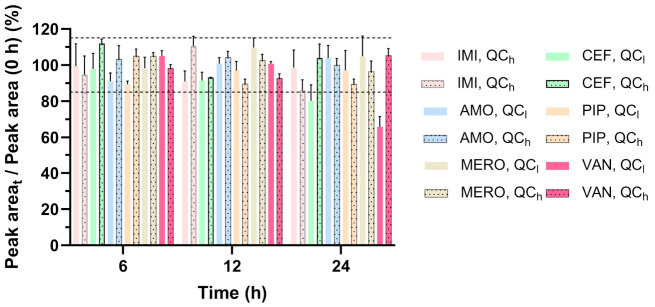
Postpreparative stability of antibiotics in autosampler at 5 °C and two concentrations (QC_l_ and QC_h_). Dashed lines represent 15% deviation.

The postpreparative stability was examined by keeping replicates of QC samples at 5 °C in autosampler. Changes in the mean peak area after 6 h, 12 h and 24 h did not exceed 13.9%, regardless of the concentration of CEF and VAN. QC_l_ samples of CEF and VAN were stable for only 12 h. The results of the stability study are shown in Figure 3.

### 2.4. Method Application

By conducting an in vitro study, we aimed to mimic in vivo conditions as closely as possible by simulating those found in septic patients (Hct 30% and Alb 20 g/L). In a similar study by C. König and colleagues [20], antimicrobial agents were dissolved in saline, albumin solution, and reconstituted blood. The prepared solutions were pumped through the CytoSorb^®^ cartridge. Unlike the aforementioned study, which did not account for the conditions of septic patients, our in vitro study is much simpler and therefore more practical for determining the binding of antimicrobial and other agents in vitro. Additionally, our experiment requires only small amounts of CytoSorb^®^ adsorber beads and blood. The chosen in vitro conditions allowed measurement of binding over 6 h, corresponding to those used in clinical practice. In the blank experiment (i.e., in the absence of antibiotics and Cytosorb^®^), haemolysis was 0.3% at baseline and 0.6% after 6 h. In the experiment with antibiotics, and in the experiment with antibiotics and CytoSorb^®^, haemolysis after 6 h increased to 0.8%. From these results, we concluded that haemolysis did not affect the course of the experiment. Albumin levels decreased by 19% after a 2 h experiment following the addition of CytoSorb^®^. The concentrations of antibiotics added to the blood were selected according to therapeutic concentrations. 

**Figure 4:**
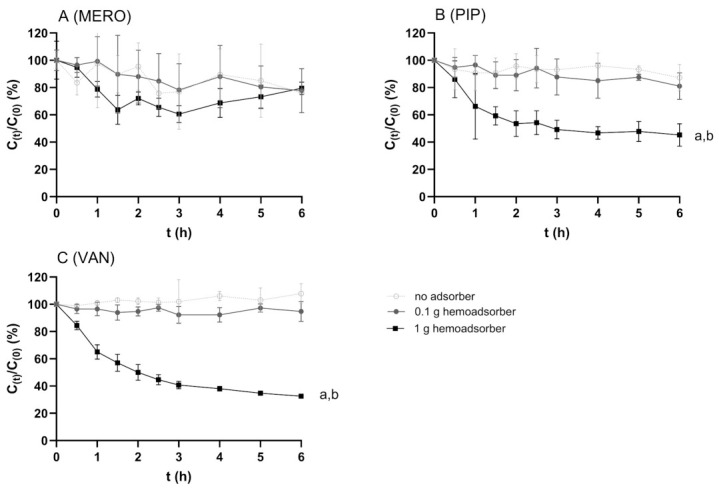
Binding of selected antimicrobial agents following the addition of different amounts of CytoSorb^®^ adsorber beads. a, b denote statistically significant differences (*p* < 0.05) between concentration ratios at 6 h under the following conditions: no CytoSorb^®^ (a) and 0.1 g CytoSorb^®^ (b).

Figure 4 shows the binding of MERO, PIP, and VAN to CytoSorb^®^ at two different haemoadsorber masses. Significant differences in binding were observed for PIP and VAN between 0.1 g and 1 g, as well as between 1 g and 0 g of CytoSorb^®^, whereas MERO showed no measurable binding.

There are scarce data available on in vitro antibiotic binding to haemoadsorbers. Published clinical [21,22,23] and in vitro [20] data on MERO are consistent with the results of our study, demonstrating negligible binding of MERO to CytoSorb^®^. Our findings are also in agreement with clinical data on VAN, indicating significant adsorption to CytoSorb^®^, which may require dose adjustment or replacement [6,7]. In contrast, clinical studies suggest that PIP does not significantly bind to CytoSorb^®^ [22,23], whereas our in vitro results indicate measurable binding. Despite this discrepancy, in vitro models provide a rapid and useful approach for estimating drug adsorption and can support the design of subsequent clinical studies. To ensure appropriate management of patients receiving concomitant therapy with CytoSorb^®^ and antibiotics, further clinical studies are warranted.

### 2.5. Limitations

This study has several limitations that should be considered when interpreting the results. First, the sample preparation procedure is relatively labour-intensive and involves the use of organic solvents, which may limit its suitability for routine laboratory applications and require appropriate safety precautions. On the other hand, the chromatographic method itself is rapid and selective, enabling reliable quantification of the investigated antibiotics in complex biological matrices.

Second, the in vitro adsorption model was performed using bovine rather than human blood due to ethical and practical approaches. Differences in plasma protein composition and other blood constituents between bovine and human blood may influence drug binding and adsorption characteristics, potentially affecting the extrapolation of the results to clinical settings.

Furthermore, the adsorption experiments were conducted in a static beaker-based system rather than in a dynamic extracorporeal circulation loop that more closely resembles clinical haemoadsorption therapy. Consequently, the entire blood volume was exposed to the CytoSorb^®^ adsorbent immediately, whereas in clinical practice, blood is continuously circulated through the cartridge. Nevertheless, we attempted to mimic septic conditions by adjusting albumin concentrations to 20 g/L and haematocrit to 30%, values commonly observed in critically ill septic patients. In addition, typical blood flow rates through the CytoSorb^®^ cartridge are approximately 200 mL/min, resulting in rapid and repeated exposure of the circulating blood volume to the adsorbent. Therefore, despite the simplified experimental setup, we believe that our model provides a reasonable approximation of antibiotic adsorption under clinically relevant conditions and allows comparative assessment of drug binding to the CytoSorb^®^ haemoadsorber.

## 3. Materials and Methods

### 3.1. Chemicals and Reagents

IMI, AMO, CEF, MERO, VAN, PIP, acetonitrile, 2-(N-morpholino)ethansulfonic acid (MES), bovine serum albumin, and bromocresol green were supplied by Sigma-Aldrich (Steinheim, Germany). IS, tetracycline chloride, was supplied by Biosynth Carbosynth (Berkshire, UK). In the in vitro experiment, bovine blood was spiked with antibiotic powder for concentrate for solution for infusion. We used Piperacillin/tazobactam Mylan 4 g/0.5 g (Viatris Sante, Canonsburg, PA, USA), Vancomycin Apta 500 mg (Apta Medica, Ljubljana, Slovenia), and Meropenem AptaPharma 500 mg (Apta Medica, Ljubljana, Slovenia). The haemoadsorbent was CytoSorb^®^ 300 (CytoSorbents Europe, Berlin, Germany). In addition, 5 M NaOH, 1 M NaOH, ethyl acetate, 70% perchloric acid, 25% ammonia water solution, and buffer solutions were purchased from Merck (Darmstadt, Germany). Phosphoric acid (85%) and dichloromethane were from Supelco (Bellefonte, PA, USA). Acetonitrile (ACN) was HPLC grade and all other chemicals were at least analytical grade. Water was purified using a Milli-Q Advantage A10 (Millipore Corp., Billerica, MA, USA). Bovine blood was obtained from the local slaughterhouse. 

### 3.2. Instrumentation

Chromatographic analyses were performed on an Agilent 1100 series HPLC system (Agilent, Waldbronn, Germany), comprising a quaternary pump solvent delivery system, an autosampler, and a diode array detector. Separation was carried out on a ZORBAX (Agilent Technologies, Palo Alto, CA, USA) Eclipse XDB C18 column (5 μm, 150 mm × 4.6 mm) coupled to a Phenomenex C18 guard precolumn (5 μm, 4 mm × 3 mm), using gradient elution. The mobile phase consisted of 0.5% phosphoric acid at pH 7 (mobile phase A) and acetonitrile with 0.5% phosphoric acid at pH 3 (1:3, *v*/*v*) (mobile phase B), with the following gradient steps (time [min], % B): (0.0, 0), (6.0, 50), (8.0, 83), (9.0, 83), (11.0, 100), (12.0, 100), (12.1, 0). The mobile phase flow rate was 1.3 mL/min. The column temperature was set to 40 °C. A 20 μL sample was injected, and analytes were detected with the diode array detector set at wavelengths of 220 nm (CEF), 230 nm (AMO, VAN, PIP), 245 nm (IMI) and 300 nm (MERO and IS).

### 3.3. Standard Solution, Calibration Standards and Quality Control Samples

A stock solution with a concentration of 1 mg/mL, as well as working solutions, calibration, and quality control (QC) samples of IMI, AMO, CEF, MERO, VAN, PIP, and IS (tetracycline), were prepared in 250 mM MES at pH 7. All solutions were freshly prepared each day and kept on ice until analysis. Calibrators for AMO and VAN were prepared at concentrations of 1, 2.5, 5, 10, 25, 50, 75, and 100 µg/mL; for IMI at 2.5, 5, 10, 25, 50, 75, and 100 µg/mL; and for CEF, MERO, and PIP at 1, 2.5, 5, 10, 25, 50, and 75 µg/mL. Similarly, QC samples of IMI, AMO, CEF, MERO, VAN, and PIP at low, medium, and high concentrations were prepared at 3, 20, and 70 µg/mL, respectively. The concentration of IS was 75 µg/mL.

### 3.4. Sample Preparation

For the calibration curve, 20 μL of the working solution of each analyte, making a total mixture of 120 μL, 20 μL of the IS solution, and 140 μL of drug-free plasma were combined. For in vitro binding analysis, 120 μL of 250 mM MES at pH 7.0, 20 μL of IS solution, and 140 μL of plasma were mixed. In the next step, 280 μL of chilled ACN was added, mixed, and centrifuged at 21,300× *g* at 4 °C for 20 min. Then, 400 μL of the supernatant was mixed with 800 μL of dichloromethane and centrifuged under the same conditions as in the previous step. After centrifugation, 90 μL of the aqueous phase was pipetted into inserts and used for analysis.

### 3.5. Method Validation

The method validation was conducted in accordance with the FDA Bioanalytical Method Validation Guidance for Industry [24]. The validated parameters—selectivity, linearity, LLOQ, accuracy and precision, carry-over, freeze–thaw stability, postpreparative and long-term stability—were determined using bovine plasma.

Selectivity was investigated by analysing six independent blank plasma samples, each assessed for any chromatographic interference at the retention times of the antibiotics and the IS.

The linearity of the method was evaluated using calibration plots constructed with calibrators over three validation days. A non-weighted linear regression analysis was applied to calculate the slopes, intercepts, and coefficients of determination by plotting the analyte–IS peak area ratios. LLOQ was defined as the lowest concentration that produced a coefficient of variation (CV) ≤ 20% and accuracy within 80–120% of the nominal, with an analyte signal response at least fivefold higher than the response in the blank sample.

Intra-day accuracy and precision were evaluated within a single day (n = 5), while inter-day accuracy and precision were assessed over three consecutive days (n = 3). Accuracy was expressed as the ratio of the determined concentration to the nominal concentration (%), and precision as the coefficient of variation (CV, %). Acceptance criteria were defined as accuracy within 85–115% of the nominal value and precision (CV) ≤ 15% for all quality control concentrations.

Carry-over was evaluated by injecting blank samples immediately after the highest calibration standard and was considered acceptable if the response in the blank sample did not exceed 20% of the analyte response at the LLOQ.

Freeze–thaw stability was assessed by subjecting QC samples to two freeze–thaw cycles, consisting of freezing at −70 °C and thawing at room temperature. After the final cycle, samples were analysed and compared with freshly prepared QC samples. Stability was confirmed if the measured concentrations were within ±15% of nominal values.

Long-term stability was evaluated by storing QC samples at −70 °C for one week, corresponding to the maximum storage duration prior to analysis. After storage, QC samples were analysed and compared with freshly prepared QC samples. Analytes were considered stable if the measured concentrations remained within ±15% of nominal values.

Postpreparative stability of QC samples in the autosampler (5 °C) was investigated by injecting the processed samples in three replicates over a period of 24 h (0 h, 6 h, 12 h and 24 h) at a temperature of 5 °C. The effect of storage conditions on the QC samples was determined with an absolute mean peak response percentage deviation ≤15% as the minimum acceptance criteria.

Partial validation, including accuracy, intra- and inter-day precision, method range, and LLOQ, was conducted in human plasma in accordance with the described criteria above.

### 3.6. In Vitro Binding of Selected Antibiotics to the CytoSorb^®^ Haemoadsorber

**Figure 5:**
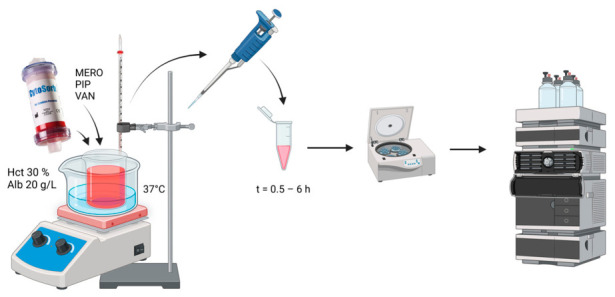
Schematic presentation of the in vitro study.

The in vitro study was conducted using bovine blood obtained from a local slaughterhouse (Figure 5). The study was approved by the Administration for Food Safety, Veterinary Sector and Plant Protection of the Republic of Slovenia (permit number U34453-63/2023/3). To mimic septic conditions, the blood albumin concentration and haematocrit were adjusted to 20 g/L and 30%, respectively. The albumin concentration in the blood was determined according to the method developed by Leonard et al. [25], which is based on spectrophotometric analysis of the bromocresol green complex with albumin. The haematocrit was determined by visually measuring the heights of plasma and blood cells in blood centrifuged in microcapillary tubes, the so-called “micro-HCT” [26]. In the experiment, we tested the binding of three different antibiotics—MERO, PIP with tazobactam, and VAN—to the CytoSorb^®^ haemoadsorbent. The antibiotic stock solutions were prepared from drug infusion powders according to the instructions in the Summary of Product Characteristics. To 100 mL of blood with adjusted albumin concentration and haematocrit, antibiotic was added to reach its maximum therapeutic concentrations (MERO = 16.0 mg/L, PIP = 80.0 mg/L, and VAN = 20.0 mg/L), followed by the addition of 0, 0.1, or 1 g of CytoSorb^®^.

Four parallel samples were prepared, each containing 100 mL of adjusted blood: three samples each containing a single antibiotic (MER, PIP or VAN), and one control sample containing all three antibiotics but without CytoSorb^®^. Samples were thermostated at 37 °C and mixed at 190 rpm for 10–15 min, followed by the addition of antibiotics. Then, 15 min after antibiotic addition, 1.5 mL aliquot of blood was collected from each beaker to determine the initial antibiotic concentrations. After 5 min, 0.1 g or 1 g of CytoSorb^®^ was added to the beakers. Next, 5 min after CytoSorb^®^ addition, serial 1.5 mL blood samples were collected at predefined time points (0, 0.5, 1, 1.5, 2, 2.5, 3, 4, 5, and 6 h) to construct the antibiotic concentration–time profiles. Samples were centrifuged for 10 min at 1800× *g* and 4 °C. The separated plasma was divided into two aliquots: one for haemolysis assessment and the other for antibiotic concentration determination. Plasma samples for antibiotic quantification were stored at −70 °C for up to one week, whereas samples intended for haemolysis and haemoglobin determination were processed on the day of the experiment. Haemolysis was evaluated at time 0 and 6 h. Haemoglobin concentrations in plasma and blood cells were measured and used to assess haemolysis. Haemoglobin concentration was determined using a method based on that reported by Frenchik et al. [27], involving the conversion of haemoglobin into a coloured product measurable spectrophotometrically at 575 nm. Antibiotic concentrations in plasma samples were determined using the validated method developed in this study.

### 3.7. Data Analysis

All data analyses were performed using Microsoft Excel 2019 MSO, GraphPad Prism 10, and IBM SPSS Statistics for Windows version 29. Multiple comparisons of drug binding across different CytoSorb^®^ masses were performed using one-way ANOVA with Bonferroni post hoc correction. Statistical significance was set at *p* < 0.05.

## 4. Conclusions

In this study, we developed and validated an analytical method that meets the required validation criteria and, additionally, established an in vitro model to assess antibiotic binding to the CytoSorb^®^ haemoadsorber using bovine blood under conditions mimicking sepsis, including haematocrit and albumin levels representative of septic patients. Using this model, we demonstrated that PIP and VAN bind to the haemoadsorbent, whereas MERO does not. Further clinical studies are needed to determine the influence of CytoSorb^®^ on antibiotic concentrations and, consequently, on dosing regimens. To appropriately manage the patients receiving concomitant therapy with CytoSorb^®^ and either VAN or PIP, we recommend the use of therapeutic drug monitoring and further investigation in critically ill patients.

## Figures and Tables

**Table 1:** Bioanalytical method validation parameters (accuracy, precision, linearity, range, and LLOQ) for the determination of selected antibiotics in bovine plasma.

**IMI**					**AMO**				
	**Accuracy (%)** **n = 5**		**Intra-Day Precision (%)** **n = 5**	**Inter-Day Precision (%)** **n = 3**		**Accuracy (%)** **n = 5**		**Intra-Day Precision (%)** **n = 5**	**Inter-Day Precision (%)** **n = 3**
QC_l_	109		12.0	14.3	QC_l_	94.1		6.1	1.6
QC_m_	104		9.88	10.1	QC_m_	108.5		14.0	12.5
QC_h_	96.5		7.65	10.1	QC_h_	97.5		3.1	2.5
Range		2.5–100.0 µg/mL			Range		1.0–100.0 µg/mL		
LLOQ		2.5 µg/mL			LLOQ		1.0 µg/mL		
Linearity		y = (0.0767 ± 7.40 × 10^−3^)x – (0.301 ± 2.81 × 10^−2^) r^2^ = 0.995–1.000			Linearity		y = (0.422 ± 4.25 × 10^−2^)x + (9.30 × 10^−2^ ± 1.55 × 10^−1^) r^2^ = 0.982–1.000		
**CEF**					**MER**				
	**Accuracy (%)** **n = 5**		**Intra-Day Precision (%)** **n = 5**	**Inter-Day Precision (%)** **n = 3**		**Accuracy (%)** **n = 5**		**Intra-Day Precision (%)** **n = 5**	**Inter-Day Precision (%)** **n = 3**
QC_l_	85.8		2.7	13.4	QC_l_	86.0		8.0	4.8
QC_m_	104.8		11.0	9.2	QC_m_	103.0		7.8	7.6
QC_h_	91.1		5.0	8.6	QC_h_	98.5		5.8	4.6
Range		1.0–75.0 µg/mL			Range		1.0–75.0 μg/mL		
LLOQ		1.0 µg/mL			LLOQ		1.0 µg/mL		
Linearity		y = (0.526 ± 5.59 × 10^−2^)x − (4.87 × 10^−3^ ± 1.11 × 10^−1^) r^2^ = 0.998–1.000			Linearity		y = (0.340 ± 5.78 × 10^−2^)x + (1.39 × 10^−1^ ± 1.32 × 10^−1^) r^2^ = 0.989–0.999		
**VAN**					**PIP**				
	**Accuracy (%)** **n = 5**		**Intra-Day Precision (%)** **n = 5**	**Inter-day Precision (%)** **n = 3**		**Accuracy (%)** **n = 5**		**Intra-Day Precision (%)** **n = 5**	**Inter-Day Precision (%)** **n = 3**
QC_l_	87.4		9.9	11.2	QC_l_	97.3		1.9	9.1
QC_m_	100.8		8.4	9.8	QC_m_	100.7		9.3	5.7
QC_h_	108.6		4.1	4.5	QC_h_	92.2		2.0	11.5
Range		1.0–100.0 µg/mL			Range		1.0–75.0 μg/mL		
LLOQ		1.0 µg/mL			LLOQ		1.0 µg/mL		
Linearity		y = (0.401 ± 6.10 × 10^−2^)x − (9.57 × 10^−2^ ± 4.96 × 10^−3^) r^2^ = 0.995–0.999			Linearity		y = (0.362 ± 4.78 × 10^−2^)x + (9.97 × 10^−2^ ± 2.10 × 10^−1^) r^2^ = 0.992–0.999		

## Data Availability

The original contributions presented in this study are included in the article. Further inquiries can be directed to the corresponding author.

## Supplementary Materials

The following supporting information can be downloaded at: https://www.mdpi.com/article/10.3390/molecules31132337/s1. Figure S1: Chromatograms of selected antibiotic at lower limit of quantification in bovine plasma. Table S1. Bioanalytical method validation parameters (accuracy, precision, linearity, range, and LLOQ) for the determination of selected antibiotics in human plasma.

## Abbreviations

ACN	acetonitrile
AMO	amoxicillin
AUC	area under the curve
CEF	cefepime
CV	coefficient of variation
ECMO	extracorporeal membrane oxygenation
FDA	Food and Drug Administration
HClO_4_	perchloric acid
HPLC	high-performance liquid chromatography
H_3_PO_4_	phosphoric acid
ICU	intensive care unit
IMI	imipenem
IS	internal standard
LC-MS/MS	liquid chromatography tandem mass spectrometry
LC-UV/Vis	liquid chromatography with ultraviolet-visible detection
LLOQ	lower limit of quantification
MERO	meropenem
MES	2-(N-morpholino)ethansulfonic acid
MIC	minimal inhibitory concentration
MP1	mobile phase 1
MP2	mobile phase 2
MP3	mobile phase 3
NaOH	sodium hydroxide
PIP	piperacillin
PK/PD	pharmacokinetics/pharmacodynamics
QC_h_	quality control sample at high concentration
QC_l_	quality control sample at low concentration
QC_m_	quality control sample at medium concentration
UV/Vis	ultraviolet-visible
VAN	vancomycin
