# Supplementary material for: Development, Validation, and Application of an HPLC Method Combined with an In Vitro Model for the Determination of Antibiotic Binding to the Haemoadsorber CytoSorb®
Source: Molecules. 2026 Jul 3;31(13):2337. doi: 10.3390/molecules31132337 (PMC13363450; doi:10.3390/molecules31132337)
Supplement: Supplementary file 1 [file molecules-31-02337-s001.zip › molecules-4360473-supplementary.pdf]

Supplementary material:

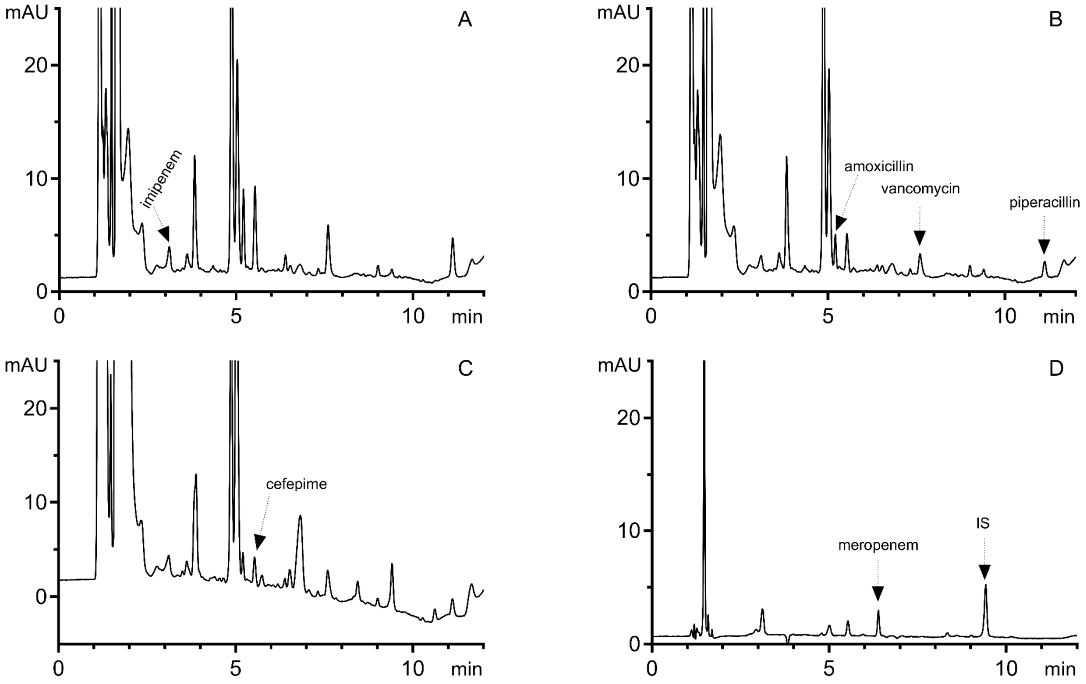

E

Figure S1: Chromatograms of selected antibiotic at lower limit of quantification in bovine plasma. (A) chromatogram obtained at 245 nm indicating imipenem peak at 3.1 min; (B) chromatogram obtained at 230 nm indicating amoxicillin, vancomycin and piperacillin peak at 5.2, 7.6, and 11.3 min, respectively; (C) chromatogram obtained at 220 nm indicating cefepime peak at 5.5 min; (D) chromatogram obtained at 300 nm indicating meropenem and internal standard peak at 6.4 and 9.4 min, respectively;

Table S1. Bioanalytical method validation parameters (accuracy, precision, linearity, range, and LLOQ) for the determination of selected antibiotics in human plasma.

IMI				AMO			
	Accuracy (%)<br>n=3	Intra-day precision (%)<br>n=3	Inter-day precision (%)<br>n=3		Accuracy (%)<br>n=3	Intra-day precision (%)<br>n=3	Inter-day precision (%)<br>n=3
QC_l	109.9	13.4	11.1	QC_l	111.6	1.7	6.7
QC_m	93.5	2.5	5.5	QC_m	110.2	2.4	6.7
QC_h	94.9	10.2	10.7	QC_h	104.3	1.2	3.3
Range	2.5 – 100.0 µg/mL			Range	1.0 – 100.0 µg/mL		
LLOQ	2.5 µg/mL			LLOQ	1.0 µg/mL		
Linearity	y = (0.0762 ± 5.12E-3)x – (3.55E-1 ± 1.27E-2)<br>r^2 = 0.986 - 0.998			Linearity	y = (0.393 ± 1.72E-1)x - (4.22E-1 ± 2.79E-1)<br>r^2 = 0.998 – 1.000		
CEF				MER			
	Accuracy (%)<br>n=3	Intra-day precision (%)<br>n=3	Inter-day precision (%)<br>n=3		Accuracy (%)<br>n=3	Intra-day precision (%)<br>n=3	Inter-day precision (%)<br>n=3
QC_l	104.4	1.3	5.7	QC_l	88.5	7.9	8.6
QC_m	100.4	3.8	10.2	QC_m	93.3	2.5	4.8
QC_h	101.9	5.2	13.3	QC_h	92.0	9.2	10.0
Range	1.0 – 75.0 µg/mL			Range	1.0 – 75.0 µg/mL		
LLOQ	1.0 µg/mL			LLOQ	1.0 µg/mL		
Linearity	y = (0.513 ± 5.34E-2)x - (4.43E-1 ± 4.33E-1)<br>r^2 = 0.998 - 1.000			Linearity	y = (0.334 ± 2.26E-2)x - (2.49E-3 ± 1.10E-1)<br>r^2 = 0.998 – 0.999		
VAN				PIP			
	Accuracy (%)<br>n=3	Intra-day precision (%)<br>n=3	Inter-day precision (%)<br>n=3		Accuracy (%)<br>n=3	Intra-day precision (%)<br>n=3	Inter-day precision (%)<br>n=3
QC_l	102.7	8.5	8.2	QC_l	92.1	8.3	14.5
QC_m	108.4	3.0	4.5	QC_m	101.2	2.2	5.6
QC_h	105.8	5.9	6.4	QC_h	107.2	9.8	10.3
Range	1.0 – 100.0 µg/mL			Range	1.0 – 75.0 µg/mL		
LLOQ	1.0 µg/mL			LLOQ	1.0 µg/mL		
Linearity	y = (0.443 ± 2.03E-2)x - (7.23E-2 ± 5.38E-2)<br>r^2 = 0.998 - 1.000			Linearity	y = (0.412 ± 2.30E-2)x + (2.07E-0 ± 1.7E-1)<br>r^2 = 0.998 – 0.999		
